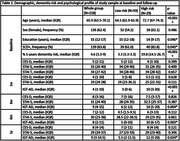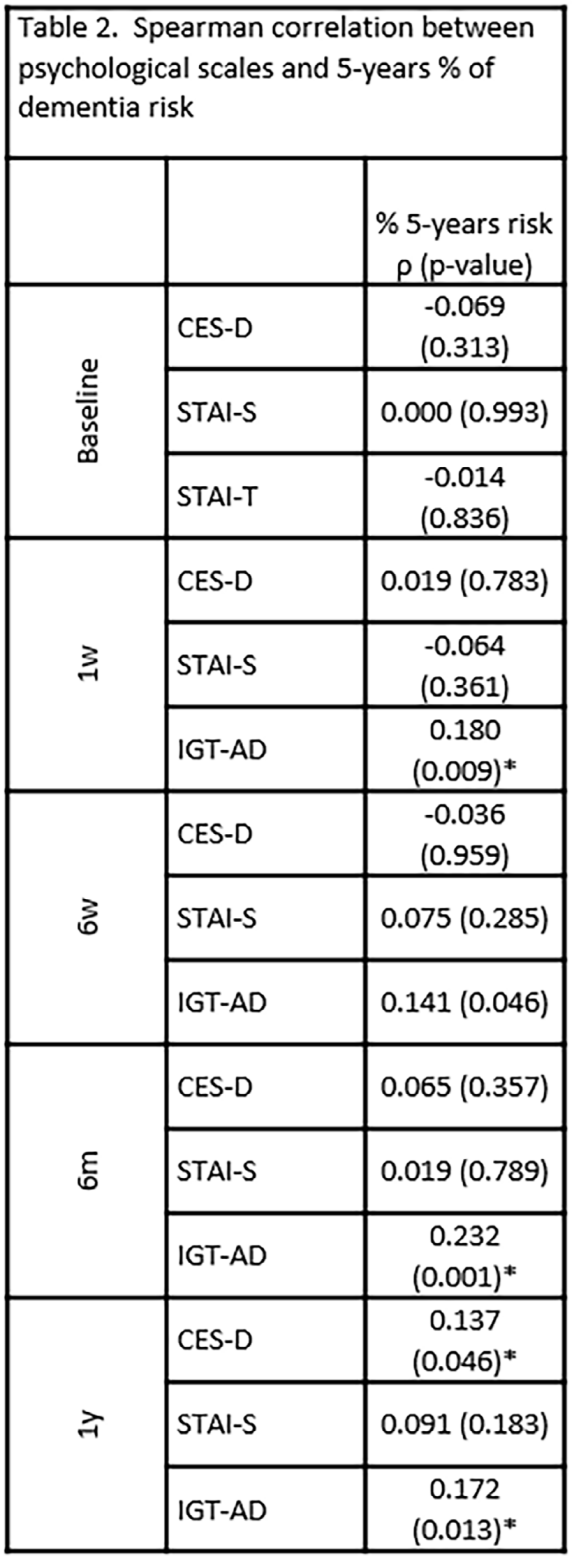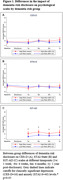# Psychological effects of personalized dementia risk disclosure: The Barcelonaβeta Dementia Prevention Research Clinic study

**DOI:** 10.1002/alz.092351

**Published:** 2025-01-09

**Authors:** Oriol Grau‐Rivera, Gonzalo Sánchez‐Benavides, Marc Suárez‐Calvet, Juan Domingo Gispert, Karine Fauria, Carolina Minguillón, Jose Luis Molinuevo

**Affiliations:** ^1^ Servei de Neurologia, Hospital del Mar, Barcelona Spain; ^2^ Centro de Investigación Biomédica en Red de Fragilidad y Envejecimiento Saludable (CIBERFES), Madrid Spain; ^3^ Hospital del Mar Research Institute (IMIM), Barcelona Spain; ^4^ Barcelonaβeta Brain Research Center (BBRC), Barcelona Spain; ^5^ Centro de Investigación Biomédica en Red Bioingeniería, Biomateriales y Nanomedicina (CIBER‐BBN), Instituto de Salud Carlos III, Madrid Spain; ^6^ Barcelonaβeta Brain Research Center (BBRC), Pasqual Maragall Foundation, Barcelona Spain; ^7^ Lundbeck A/S, Copenhagen Denmark

## Abstract

**Background:**

Approximately 35‐40% of new patients at memory clinics are cognitively intact individuals concerned about their dementia risk. The demand for personalized risk profiling and preventive strategies for those at higher dementia risk is in need of innovative infrastructures. However, disclosing dementia risk estimates raises concerns about potential negative emotional impacts. To address this challenge, the Barcelonaβeta Dementia Prevention Research Clinic, was established to evaluate the risks and benefits of novel prevention infrastructures and the potential impact of dementia risk disclosure. Here we focus on the psychological effects of personalized dementia risk disclosure on cognitively unimpaired individuals with subjective cognitive decline (SCD).

**Method:**

Participants aged 60‐80 with cognitive complaints were pre‐selected using web‐based algorithms. Eligible participants were invited to an in‐person visit and a clinical diagnosis was established after neuropsychological, blood tests and magnetic resonance imaging assessments. Dementia risk over 5 years (%risk) was calculated and disclosed with personalized prevention plans. Impact on depression (CES‐D), anxiety (STAI), and test‐related distress (IGT‐AD) was assessed up to 12 months post‐disclosure. SCD participants were categorized into “high dementia risk” (HDR, %risk>10.8) and “low dementia risk” (LDR, %risk<3.6) groups, based on previous epidemiological evidence. Wilcoxon rank‐sum tests and Spearman’s rank correlation analyzed between‐group differences and correlations at different time points.

**Result:**

The percentage of 5‐years dementia risk was disclosed to 192 participants with SCD. Test‐related distress (IGT‐AD) was consistently higher in HDR (N = 25) vs. LDR (N = 55), while CES‐D and STAI showed no significant differences between groups (Table 1, Figure 1). Similar results were observed in correlation analyses for the entire sample (Table 2). Median CES‐D and STAI‐S values remained below clinically relevant cutoffs for depression and anxiety across all visits.

**Conclusion:**

Disclosure of dementia risk heightened test‐related distress but did not manifest clinically relevant negative effects in anxiety and depression scales. The potential impact of this psychological response on promoting engagement in preventive behaviors warrants further exploration.